# Supplementary material for: Charting the N-Terminal Acetylome: A Comprehensive Map of Human NatA Substrates
Source: Int J Mol Sci. 2021 Oct 2;22(19):10692. doi: 10.3390/ijms221910692 (PMC8509067; doi:10.3390/ijms221910692)
Supplement: Supplementary file 1 [file ijms-22-10692-s001.zip › Figure_S1.pdf]

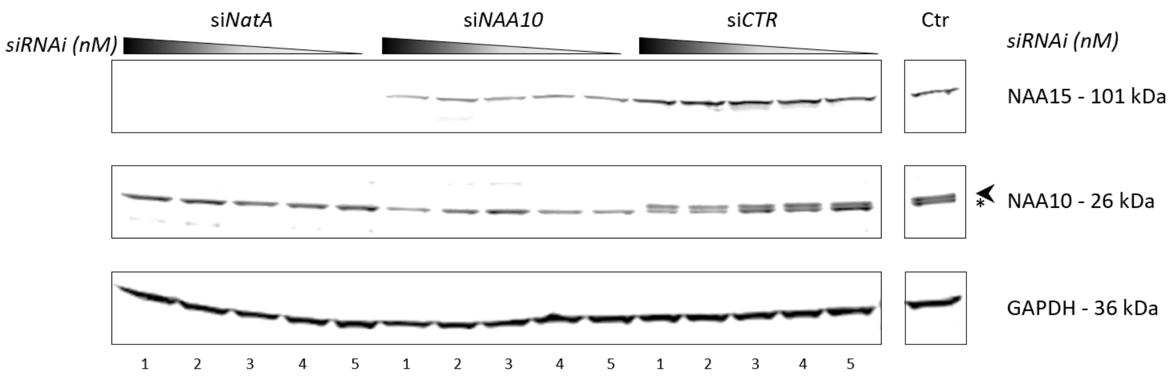

Figure S1| Optimization of si*NatA*-knockdown conditions in human A-431 cells. siRNA transfections were performed using HiPerFect (Qiagen) and varying concentration (5-100 nM) of non-targeting siRNA (si*CTR*), si*NAA10* or si*NAA10*/si*NAA15* pool (si*NatA*). Cells were transfected 24 hours after seeding, re-transfected under identical conditions 48 hours after initial transfection and harvested 96 hours post initial siRNA transfection. Non-transfected cells (Ctr) served as an additional control. Blots were probed with anti-NAA10 and anti-NAA15 to assess levels of endogenous NAA10 (26 kDa) and NAA15 (101 kDa). Next to GAPDH (36 kDa), the asterisk indicates a non-specific band that serve as loading control. Numbering from 1 to 5 correspond to siRNA concentrations of 100, 50, 20, 10 and 5 nM, respectively.
