# Supplementary material for: Charting the N-Terminal Acetylome: A Comprehensive Map of Human NatA Substrates
Source: Int J Mol Sci. 2021 Oct 2;22(19):10692. doi: 10.3390/ijms221910692 (PMC8509067; doi:10.3390/ijms221910692)
Supplement: Supplementary file 1 [file ijms-22-10692-s001.zip › Figure_S2.pdf]

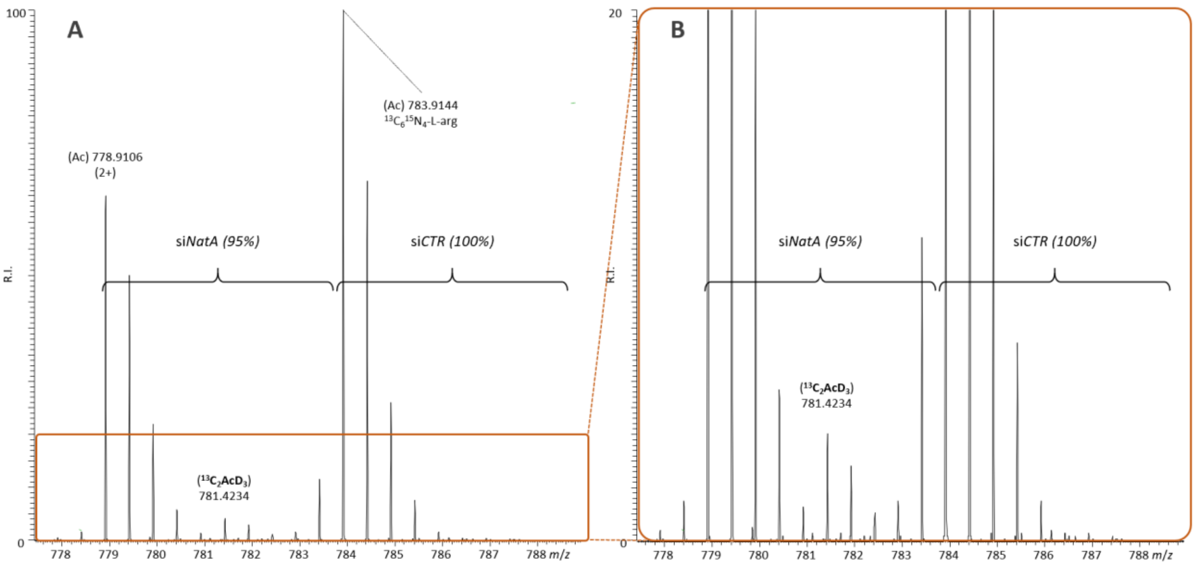

Figure S2| The effect of si*NatA* knockdown on the Ala- starting NatA type N-terminus of THUMP domain-containing protein 1. (**A**) MS spectrum of the N-terminus originating from THUMP domain-containing protein 1 ([2]AAPAQQTTQPGGGKR[16]) is shown. The peptide was fully Nt-acetylated in the control setup while being only partially Nt-acetylated (95%) in the si*NatA* knockdown sample. Since in this representative example, clearly two isotopic envelopes are discernible in the si*NatA* setup versus the exclusive occurrence of isotopic envelope corresponding to the Nt-acetylated heavy labelled peptide in the s*iCTR* setup (zoomed panel **B**), similar examples with a minimum difference of 5% in the degree of NTA are additionally considered as being affected by si*NatA* knockdown (Supplemental Tables S1-2 and 'Material and Methods').
